# *Manilkara zapota*: From Phytochemistry to Therapeutics, and Relevance to Food Industries

**DOI:** 10.3390/foods15111968

**Published:** 2026-06-02

**Authors:** Ayesha Siddiqa, Adnan Amjad, Jasenka Gajdoš Kljusurić, Zafarullah Muhammad, Shehnshah Zafar, Muhammad Khurram Afzal, Muhammad Maaz, Muhammad Israr, Syeda Khimar Zahra Kazmi, Naveed Ahmad, Asad Abbas

**Affiliations:** 1 Faculty of Food Science and Nutrition, Bahauddin Zakariya University, Multan 60800, Pakistan; asiddiqa442@gmail.com (A.S.); shah8942123@gmail.com (S.Z.); khurram.afzal@bzu.edu.pk (M.K.A.); maazm4158@gmail.com (M.M.); misrarjutt139@gmail.com (M.I.); khimarzahra@gmail.com (S.K.Z.K.); 2 Faculty of Food Technology and Biotechnology, University of Zagreb, Pierottijeva 6, 10000 Zagreb, Croatia; 3 College of Agriculture and Food Engineering, Baise University, Baise City 533000, China; zafwahla@bsuc.edu.cn; 4 Multan College of Nutritional Sciences, Multan Medical and Dental College, Multan 60000, Pakistan; navidahmad.ft@gmail.com

**Keywords:** *Manilkara zapota*, nutritional composition, phytochemistry, therapeutic potential, anticancer, food applications

## Abstract

*Manilkara zapota* (*M. zapota*), commonly known as sapodilla, is a tropical fruit recognized for its nutritional value and diverse phytochemical composition. This review critically summarizes recent evidence (2013–2026) regarding the phytochemistry, biological activities, safety profile, and food industry relevance of *M. zapota*, using literature retrieved from Google Scholar, PubMed, Scopus, Web of Science, Science Direct, and other scientific databases. Different parts of the plant, including its fruits, leaves, seeds, and bark, contain a wide range of bioactive compounds, such as gallic acid, caffeic acid, quercetin, catechin, myricetin, kaempferol, β-sitosterol, stigmasterol, alkaloids, tannins, saponins, triterpenes, and glycosides. Experimental studies have demonstrated antioxidant, anti-inflammatory, antimicrobial, gastroprotective, glucose homeostasis, and antiproliferative activities associated with these phytochemicals. Mechanistically, *M. zapota* extracts have been reported to modulate oxidative stress markers, inflammatory mediators, apoptotic pathways, and lipid metabolism-related enzymes in in vitro and animal studies. Available toxicological evidence suggests that certain extracts were well tolerated under specific experimental conditions; however, further standardized safety assessments and clinical investigations remain necessary. In addition to its pharmacological relevance, *M. zapota* has potential applications in functional foods and food processing industries, including jams, jellies, spreads, fruit bars, beverages, and nutraceutical formulations. Overall, *M. zapota* represents a promising underutilized plant with potential relevance for food, nutraceutical, and future biomedical applications.

## 1. Introduction

Nature provides a diverse range of plants that contribute to human nutrition and overall health. Different plant parts, such as roots, leaves, stems, seeds, bark, flowers, and fruits, contain bioactive constituents with functional and properties that may help support health and reduce the risk of non-communicable diseases [1]. Fruits are an important source of vitamins, minerals, dietary fiber, and phytochemicals such as phenolic acids, flavonoids, carotenoids, anthocyanins, tannins, saponins, and terpenoids. These compounds have been associated with antioxidant, anti-inflammatory, and metabolic regulatory activities that may contribute to the prevention and management of disorders, such as type 2 diabetes mellitus (T2DM), obesity, cardiovascular diseases, gastrointestinal disorders, hepatic diseases, and certain cancers [2,3]. The American Dietetic Association (ADA) recommends regular fruit consumption as a part of a balanced diet to support nutritional adequacy and overall well-being [4]. However, rapid industrialization and urbanization have significantly altered dietary habits and lifestyles. Increased consumption of highly processed and ready-to-eat food, combined with sedentary behavior, has contributed to oxidative stress, chronic inflammation, and the growing prevalence of metabolic diseases [5].

Consequently, interest in functional foods and nutraceuticals derived from plant-based bioactive compounds has increased considerably in recent years. These products are being investigated for their potential role in improving nutritional quality and supporting health maintenance [6]. Several nutritionally valuable but underutilized plant species, including buckwheat, sorghum, millet, amaranth, quinoa, taro, purple yam, grass pea, chayote, and linseed, remain insufficiently explored, despite their potential food and pharmacological relevance. Limited utilization of such crops may contribute to challenges related to food security, nutritional sustainability, and dietary diversity, particularly in developing regions. One such underutilized tropical plant is *Manilkara zapota* (*M. zapota*), commonly known as sapodilla, sapota, chiku, naseberry, or chico sapote [7,8]. *M. zapota* is recognized for its sweet edible fruit and its diverse phytochemical composition, which has attracted attention in food, nutraceutical, and pharmacological research. Traditionally, different parts of *M. zapota* have been used in folk medicine for various purposes. The seeds have been described as having diuretic and tonic properties, whereas the bark has traditionally been used for its astringent and febrifuge characteristics [9,10,11,12]. In addition, extracts obtained from the fruits and leaves have been investigated for antioxidant, anti-inflammatory, antimicrobial, antiproliferative, antipyretic, and gastroprotective activities in experimental models [13,14,15,16].

Previously published reviews on *M. zapota* have mainly focused on selected aspects, including nutritional composition, phytochemistry, or pharmacological activities. However, a comprehensive and updated evaluation integrating recent findings on phytochemical composition, mechanistic biological activities, safety considerations, and food industry relevance remains limited. Therefore, the present review critically summarizes studies published between 2013 and 2026 regarding the nutritional profile, phytochemical constituents, experimental biological activities, safety, and industrial applications of *M. zapota*. Particular emphasis is placed on the relationship between its phytochemical composition and potential food-relevant biological properties, including antioxidant, anti-inflammatory, glucose homeostasis, and gastroprotective activities. In addition, this review discusses the potential relevance of *M. zopata* in the development of functional foods and value-added products, including jams, spreads, beverages, fruit bars, and nutraceutical formulations. Pharmacological findings included in this review are intended to describe experimental evidence and possible biological mechanisms associated with *M. zapota* phytochemicals. Overall, this review aims to provide an updated and critical overview of the nutritional, phytochemical, biological, and industrial significance of *M. zapota*, while highlighting current research gaps and future perspectives.

## 2. Search Methodology

**Figure 1:**
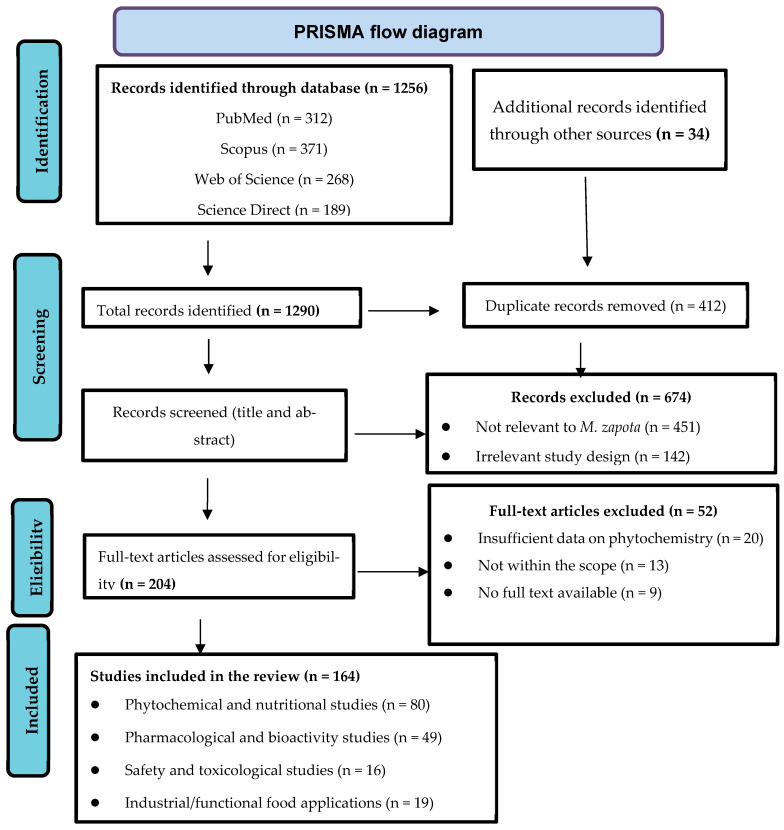
Schematic diagram for study selection.

This review assembled and critically analyzed published literature on *M. zapota* from Google Scholar, PubMed, Science Direct, Web of Science, Scopus, and Directory of Open Access Journals from 2013–2026, facilitating an update on the evidence regarding its phytochemistry, mechanistic pharmacology, safety, and industrial applications by using Boolean operations (and/or), MeSH terms, and keywords, such as sapodilla, *M. zapota*, botanical description of *M. zapota*, nutritional or proximate or micronutrient or macronutrient composition of *M. zapota*, phytochemistry or phytochemical profile of *M. zapota*, antioxidant potential of *M. zapota*, anticancer activity and *M. zapota* or sapodilla, anti-inflammatory or inflammation and *M. zapota*, cardioprotection by *M. zapota*, hepatoprotective or liver or liver cirrhosis or liver fibrosis and *M. zapota*, diabetes or glucose metabolism-related activity and *M. zapota*, aging or antiaging and *M. zapota*, safety and toxicity of *M. zapota*, and industrial applications of *M. zapota*. The schematic diagram for study selection is shown in Figure 1.

## 3. Botanical, Geographical, and Morphological Description

**Figure 2:**
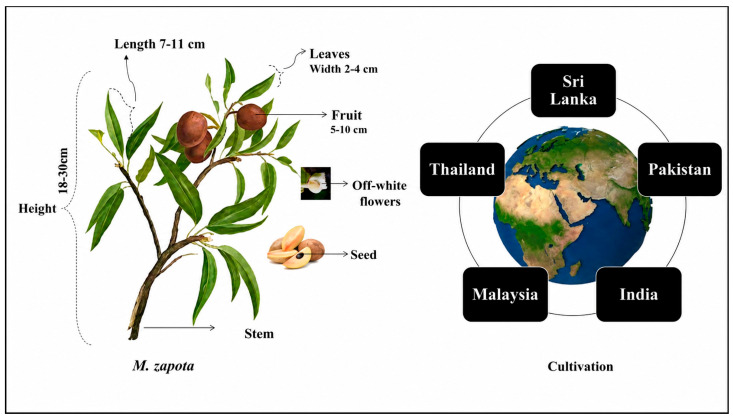
Geographical and botanical description of *M. zapota*. The globe is showing the major cultivating countries that produce *M. zapota* annually.

The plant is renowned for alternative nomenclatures, such as chiku, nasberry, and sapota, across different regions. Sapodilla, belonging to the *Sapotaceae* family, is usually cultivated in the tropical and subtropical areas of the world (Figure 2), particularly in Pakistan, India, Sri Lanka, Bangladesh, the Philippines, Indonesia, Malaysia, and Thailand [17,18]. Climatic, environmental, and irrigation fluctuations do not disturb its growth. As a result, it grows throughout the year, particularly from June to August, from October to December, and in March. However, it requires ~5–8 years to achieve maximum growth and pubescence [9]. The young evergreen tree, *M. zapota*, has a pyramidal canopy that makes it an air-resistant tree. Its milky latex “chicle” is exuded from all the parts, reaching a height of ~18–30 m [14,19]. The elliptic or oblong young leaves are light-green to pink, 7–11 cm length, and 2–4 cm width, and become shiny and dark green upon reaching puberty. Additionally, its leaves are alternate and spirally arranged at the tips of shoots. The off-white and greenish flowers are small, bell-like, and solitary, comprising three brown, hairy outer sepals and three inner sepals with pale-green corolla. However, the yellow, brownish fruits are round to oval with rough skin, with a diameter of 5–10 cm, while their brown and black, long, oval, and shiny seeds are 2 cm long [14].

## 4. Nutritional and Phytochemical Composition

Indigenous populations consume fruits, particularly sapodilla, to meet their nutritional requirements due to the ample carbohydrates, proteins, fat, fiber, vitamins, and minerals that they contain. These nutrients play a role in preventing and managing nutrient deficiency disorders [20]. The fruit accounts for macronutrients—~14–20% carbohydrates, 11–15% sugars, 7–9% reducing sugars, 0.52–0.76% proteins, 0.6–1.1% fats, and 60–69% moisture. Regarding micronutrients, 0.4–0.6% ash, 0.01–0.21% dietary fibers [21], vitamin A (60 IU), vitamin C (14.7 mg), riboflavin (0.020 mg), niacin (0.200 mg), pantothenic acid (0.2528 mg), vitamin B6 (0.037 mg), folate (14 μg), iron (2 mg/100 g), potassium (193 mg/100 g), phosphorous (21 mg/100 g), calcium (96 mg/100 g), magnesium (12 mg/100 g), sodium (12 mg/100 g), selenium (0.6 μg/100 g), copper (1.7 μg/g), manganese (1.5 μg/g), and zinc (1.0 μg/g) are also present [18,22,23,24].

**Figure 3:**
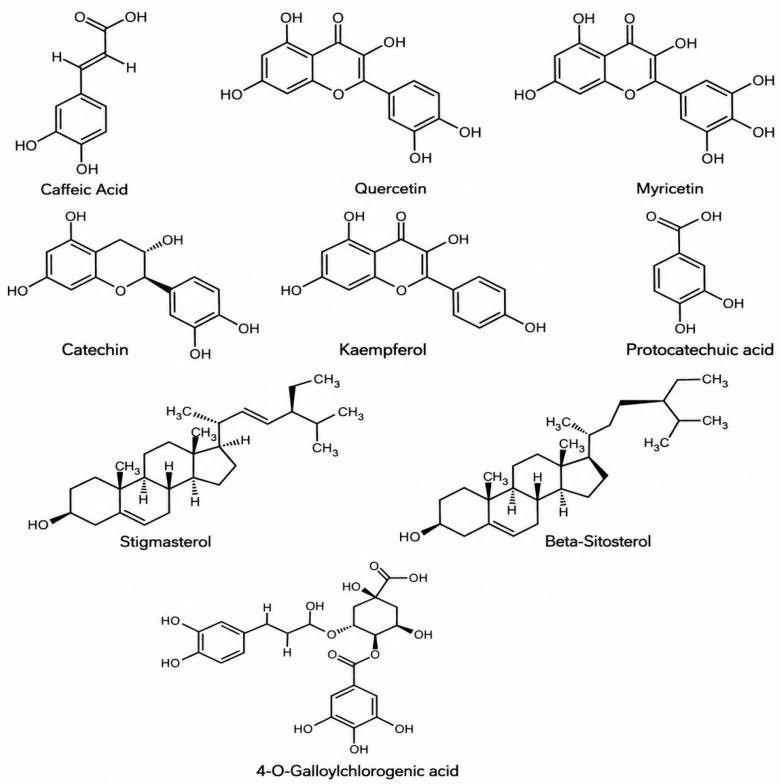
Chemical structures of bioactive compounds present in *M. zapota*.

The therapeutic constituents (phytochemicals) extracted from plants have health-promoting and disease-ameliorating properties. Various phytochemicals have been identified and isolated from leaves, fruits, seeds, and bark of *M. zapota*. The nutritional composition and bioactive compounds in different parts of *M. zapota* are documented in Table 1. Its leaves contain hydrocarbons [n-triacontane (46.5–49.5%), n-octacosane (18.3–23.8%), β-sitosterol (1.47–3.41%), and stigmasterol (1.09–2.6%)], saturated fatty acids (SFA_S_) (6.8–9.9%), unsaturated fatty acids (UFAs) (27.8–32.1%), and polyunsaturated fatty acids (PUFAs) (14.5–17.8%). The most important of them are oleic (10.7–13.9%), linolenic (8.3–10.1%), linoleic (3.3–5.94%), oleanolic, palmitic, and stearic acids [25]. Moreover, lupeol-3-acetate, myricetin-3-O-α-L-rhamnoside, caffeic acid, and apigenin-7-O-α-L-rhamnoside, alongside other flavonoids, alkaloids, saponins, tannins, triterpenes, and cardiac glycosides, were also identified from the leaf extract [26]. The methanolic leaf extracts exhibit a total phenolic content (TPC) and a total flavonoid content (TFC) of 194.04 mg/g and 35.53 mg/g, respectively [27]. Furthermore, fruit constitutes phytochemicals i.e., cyanogenic glycoside, terpenoids, methyl 4-O-galloylchlorogenate, 4-O-galloylchlorogenic acid, methyl chlorogenate, dihydromyricetin, quercetin, myricitrin, kaempferol, catechin, epicatechin, gallocatechin, gallic acid, protocatechuic acid, 4-O-galloylchlorogenic acid, and methyl-4-O-galloylcholorogenate (Figure 3) [13,14,28,29,30,31]. Additionally, the seeds and bark are abundantly equipped with alkaloids, flavonoids, saponins, tannins, and phenolic compounds, including D-quercitol, saccharose, Β-amyrin, oleanolic acid, lupeol, betulinic acid, and isoprenoids [32,33,34,35,36,37].

*M. zapota* has a high medicinal value due to its phytochemical composition, which varies significantly in the fruit, seed, leaves, pulp, and juices. The fruit contains significant amounts of TPC (20–85 mg GAE/g) and TFC (34.9 mg QE/g) and has high antioxidant activity (DPPH 61.3%, FRAP 540 µmol Fe^2+^/g, ABTS 65.2) [21,24]. The seeds are rich in oil (18–20%), such as oleic acid (40–50%), and have high antioxidant potential (DPPH 89.5%, FRAP 1385 µmol Fe^2+^/g, ABTS 91.2%), with gallic acid (0.065 mg/g) [25,27] and epigallocatechin (0.034 mg/g) as key bioactive compounds [29,33]. The leaves are rich in vitamin C (20–60 mg/100 g), have strong antioxidant activity (DPPH 93.8%, FRAP 1640 µmol Fe^2+^/g, ABTS 95.4%), and contain kaempferol (8.46 mg/g), S-ribosyl-L-homocysteine (RT: 0.672 min, abundance: 2.309 × 10^5^) and other bioactive compounds [27,29]. The pulp/juice of *M. zapota* contains natural sugars (14.7%), dietary fiber (5.3%), vitamin C (6–25 mg/100 g), and bioactive compounds (phenolics 2–12 mg GAE/g, flavonoids 28.6 mg QE/g). The antioxidant activity of the pulp is moderate (DPPH 52.7%, FRAP 410 µmol Fe^2+^/g, ABTS 58.9%) [18,29,31]. The concentration of antioxidants and bioactive compounds of the leaves and seeds is considerably higher than that of the fruit and seeds, especially flavonoids and sphingolipids. The variation in phytochemical levels among different parts of the plant suggests that its different phytochemicals may offer different benefits in various applications, ranging from antioxidant applications to therapeutic ones.

The nutritional and phytochemical profile of *M. zapota* shows significant variability based on the cultivar, maturation stage, geographic origin, and processing methods, factors that play a significant role in determining its use in food systems. Fully ripe fruits tend to have higher amount of reducing sugars, carotenoids, and aroma volatiles, while unripe fruits have higher levels of tannins, which impart astringency and decrease palatability. The genotype, environmental conditions, and soil composition also affect the distribution of phenolic acids, flavonoids, triterpenoids, and phytosterols among leaves, fruit pulp, peel, seeds, and bark. Phytochemical profiles have also been reported to depend on the extraction solvent and analytical method, with polar solvents optimally suited for the extraction of phenolics and nonpolar solvents for sterols and lipophilic constituents.

## 5. Traditional Applications

Its traditional use in the primary healthcare settings was to manage multiple prevailing disorders. The decoction of immature fruit has was used as an antidiarrheal, diuretic, febrifuge, and tonic agent. In contrast, the decoction of its leaves reduces the severity of diarrhea, cough, cold, fever, pain, hemorrhage, healing wounds, and ulcers [38]. Moreover, the seed and bark extracts relieve kidney and bladder stones to improve renal and bladder performance, in addition to being used as astringents [9,39,40]. The white, gooey chicle found in the tree’s bark was used in dental surgery, gum production, and pulmonary disease management [9,41].

## 6. Food-Relevant Bioactivity and Evidence Limitations

Plant parts, maturity, extraction solvent, dose, model, and endpoint measurement may vary between studies, thus limiting direct comparison between studies. The antioxidant, anti-inflammatory, glucose metabolism-related, lipid metabolism-related, digestive support, and antiproliferative results are then experimental data that are used food functionality research. The pharmacological evidence was summarized by plant part, extract type, experimental model, dose/concentration, endpoint, and evidence strength for clarity. This encompasses a structure that does not just store information, but allows recognition of a set of findings relevant to further development of food, formulation of nutraceuticals, and future clinical studies. Table 2 illustrates the presence of multiple bioactive compounds and their pharmacological effects.

### 6.1. Antioxidant Activity

Oxidative stress induces multiple disorders by disrupting the homeostasis between free radicals and antioxidants. Factors such as higher fatty food consumption, environmental contaminants, and poor dietary and lifestyle practices promote oxidative stress and stress-induced disorders [53]. Moreover, reduced levels of antioxidative enzymes such as superoxide dismutase (SOD), catalase (CAT), glutathione (GSH), glutathione peroxidase (GPx), and glutathione reductase in oxidative stress disorders are observed [54]. The antioxidant potential of *M. zapota* fruit extract obtained using hydroalcoholic means, petroleum ether, or distilled water was assessed by assessing ferric thiocyanate, hydrogen peroxide (H_2_O_2_) scavenging, and xanthine oxidase, which revealed that both fruit and leaf extracts had a dose-dependent effect on H_2_O_2_ scavenging (IC_50_ = 42 µg/mL and IC_50_ = 54 µg/mL), ferric thiocyanate (IC_50_ = 76 µg/mL and IC_50_ = 89 µg/mL), and xanthine oxidase (IC_50_ = 70 µg/mL and IC_50_ = 41 µg/mL) [9,55]. Moreover, the free radical-reducing potential of the leaves’ extract demonstrated DPPH (~86%) and FRAP with the highest concentration, i.e., 100 µg/mL [49,56]. Later, the antioxidant potential of petroleum ether, ethyl acetate, n-butanol, and water extracts of *M. zapota* leaves revealed the highest antioxidant potential of ethyl acetate extracts, with IC_50_ values of 0.019 mg/mL (ABTS and DPPH) and 0.5 mg/mL for the ABTS assay [25]. Moreover, the ethanolic leaf extract revealed its antioxidant potential by showing an IC_50_ of 16.4 µg/mL [57].

The bioactive compounds found in the different parts of *M. zapota* are responsible for its antioxidant potential [14,58]. Bioactive components from the bark of *M. zapota* include 6-hydroxyflavanone, dihydrokaempferol, and 3,4-dihydroxybenzoic acid, which protect against ROS [26]. Similarly, taraxerol methyl ether, lupeol acetate, taraxerone, taraxerol, spinasterol, 3,4-dihydroxybenzoic acid, 6-hydroxyflavanone, and (+)-dihydrokaempferol were identified and isolated from the bark, with free-radical scavenging potential determined. It has been found that spinasterol has the highest neutralizing potential, shown by DPPH (IC_50_ = 93 µM) and ABTS (IC_50_ = 921 µM). Moreover, (+)-dihydrokaempferol demonstrated relatively higher FRAP values (6.23 M) than other compounds [26]. Moreover, deep eutectic solvents (DESs), such as an acetone–water mixture (AWM), glyceline, alaline, maline, maloline, oxaline, ethaline, and reline, were used to evaluate the antioxidant profile. The results showed that maloline has the highest antioxidant activity, as revealed by ABTS (68.4 µmol AAE/g DW), DPPH (84 µmol AAE/g DW), and FRAP (63 µmol AAE/g DW), as compared to other solvents [34]. Furthermore, the antioxidant potential of the ethanolic extract of *M. zapota* was revealed by exhibiting a percentage inhibitions of 27.8% (ABTS for 7 min), 31.9% (DMPD for 10 min), and 33% (DPPH for 30 min). The antioxidant potential of ripe *M. zapota* was demonstrated by its ability to neutralize superoxide (O_2_^−^), hydrogen peroxide (H_2_O_2_), hydroxyl (OH-), and nitric oxide (NO) [57]. Moreover, the aqueous fruit extract of *M. zapota* scavenges ROS and RNS, as revealed by DPPH assay (IC_50_ = 48 µg/mL) [58].

The bioavailability and metabolic disposition of the key phytochemicals found in *M. zapota*, such as phenolic acids (gallic, caffeic, and chlorogenic acids), flavonoids (quercetin, catechin, and myricetin), and triterpenoids (lupeol and ursolic acid) vary significantly and profoundly affect the physiological activity of the compounds [9]. More complex flavonoids are poorly absorbed in the small intestine but are extensively metabolized by gut microflora and enzymes to generate bioactive metabolites. Phenolic acids are relatively well absorbed in the small intestine. Lipophilic compounds like triterpenoids and phytosterols generally have low water solubility, but, when taken in lipid-rich food matrices, the water solubility may increase, resulting in improved bioavailability [59,60]. Ripening, processing, and storage conditions also influence the bioaccessibility of these compounds by changing their stability and release from plant matter. Given the various in vitro assays that show strong antioxidant and anti-inflammatory effects, the physiological relevance and bioavailability of *M. zapota* should not be overlooked when investigating its use in functional foods and nutraceutical applications [61]. A comparative study was conducted to investigate the antioxidant potential of *M. zapota* fruits and leaves. The results revealed that the aqueous fruit extract has a higher free radical-scavenging potential than leaf extract due to flavonoids and phenolic compounds [62]. Moreover, the antioxidant potency of crude and ethyl acetate extract was determined by conducting an ABTS assay, which showed that ethyl acetate extract (43 × 10^3^ μM Trolox/g) has a relatively higher free radical-scavenging potential as compared to crude extract (22 × 10^3^ μM Trolox/g) [63]. Afterwards, the free radical-scavenging potential of the peel was assessed by preparing extracts in different solvents, e.g., ethanol, acetone, n-hexane, chloroform, and water, via DPPH and H_2_O_2_ assays. The results revealed that the ethanolic extract has higher H_2_O_2_ (65% inhibition at 50 µg/mL with IC_50_ = 32.6) and DPPH (88% inhibition at 1 µg/mL with IC_50_ = 0.34) scavenging potential than other extracts [64]. The antioxidant activity modulates the fundamental pharmacological attributes, such as anticancer, anti-inflammatory, and cardioprotective properties. Although many studies validated the antioxidant potential, the available evidence is mostly obtained from in vitro assays, such as DPPH, ABTS, and FRAP. Doubtless, these assays aid in the preliminary screening, but they do not completely indicate the biological antioxidant activity. Therefore, standardized protocols and further in vivo studies are required to prove the antioxidant properties and therapeutic potential of *M. zapota*.

### 6.2. Anticancer Activity

Cancer, the metastasis of abnormal and affected cells, is the leading cause of mortality, with ~20 million global incidences [65]. Multiple factors, such as poor dietary choices, reduced physical activity, environmental contaminants, compromised immune system, elevated exposure to infections, and genetic mutations, promote the propagation of cancerous cells [66,67]. Genes, i.e., RAS, MYC, BCL-2, HER2/neu, SRC, and BCR-ABL, promote the metastasis of affected cells, compromising life quality [68]. Natural therapeutic candidates are being researched as a novel and effective alternate to chemotherapeutic drugs.

Medicinal plants and fruits, specifically *M. zapota*, exhibit natural antiproliferative properties [69]. Hence, the hydroalcoholic extract of *M. zapota* protected against the propagation of a skin cancer (A431) cell line, with the highest cytotoxic effect at 346.5 µg/mL [54]. Phytochemicals such as taraxerol methyl ether, lupeol acetate, taraxerone, taraxerol, spinasterol, 3,4-dihydroxybenzoic acid, 6-hydroxyflavanone, and (+)-dihydrokaempferol, derived from the bark, showed cytotoxic potential against breast (BT474), lung (Chango-K1), liver (HepG2), gastric (KATO-III), colon (SW620), and human diploid lung fibroblastic (WI-38) cell lines. Spinasterol has the highest cytotoxic potential against BT474, HepG2, KATO-III, SW620, and WI-38, with respective IC_50_ values of 9 µM, 10.8 µM, 13.7 µM, 33 µM, and 9.8 µM, while higher cytotoxicity (IC_50_ = 12.3 µM) was exhibited by (+)-dihydrokaempferol against the Chago-K1 cell line [26]. The antimetastatic activity and underlying mechanism of aqueous leaf extract (ALE) were evaluated against human colorectal cancer (HT-29) cells via MTT assay. The results revealed that administering ALE (21, 42, and 84 µg/mL) for 3 days profoundly decreased cell viability and inhibited the Wnt/β-catenin pathway. Moreover, it modulated the apoptotic pathway, caspase-3, caspase-8, catalase, GSK-3β (glycogen synthase kinase 3β), AXIN1, adenomatous polyposis coli (APC), and casein kinase 1 (CK1) expression levels to induce cytotoxicity against the HT-29 cell line [70]. Additionally, the antiproliferative potential of hexane, methanol, and ethyl acetate extracts from leaves was investigated by conducting an MTT assay with human lung adenocarcinoma cells, which revealed that the ethyl acetate extract exerted the maximum growth inhibition (~45%) [71].

Podder et al. [72] conducted an in vivo study to evaluate the antimutagenic potential of *M. zapota* seed lectin against Ehrlich ascites carcinoma and human breast cancer (MCF-7) cell lines in Swiss albino rats. MTT assay revealed dose-dependent cell death, with the highest levels of growth inhibition (21.6% and 51.2%, respectively) at the 80 µg/mL concentration. Nanotechnology, including nanoemulsions and nanoparticles, has emerged as an effective management approach in multiple disorders, i.e., cancers, due to the targeted delivery of the drug, which promotes the absorption of the drug or supplement. The anticancer activity of copper nanoparticles of leaf extract was assessed in MCF-7 and Vero cells, which revealed that copper nanoparticles attenuated cancer cell proliferation with IC_50_ values of 53.8 µg/µL and 883 µg/µL, respectively [73]. Similarly, silver nanoparticles prepared from the leaf extract were effective against the HCT116, HeLa, and A549 cell lines. The nanoparticles suppressed tumor growth by inducing apoptosis, elevating ROS production, and modulating the expression of apoptotic genes, such as caspase-3, caspase-8, caspase-9, and Bax [74]. The anticancer potential of plants and compounds is improved when two or more plants and compounds are applied in combination. A study conducted by [75] demonstrated that the cytotoxic potential of *M. zapota* increased when supplemented with *Curcuma longa*. Similarly, durian and *M. zapota* synergistically modulated chemotherapeutic-induced cytotoxicity by downregulating caspase-3, caspase-9, Fas, and XIAP expression [76]. Conclusively, the results of *M. zapota* extracts and their derived compounds showed cytotoxicity against different cancer cell lines in in vitro studies. Furthermore, these studies are different in terms of extraction methods, concentrations, and experimental conditions, resulting in difficulty in identifying standard effective doses. These gaps should be filled by directing future research towards mechanistic research, pharmacokinetics, and disease-specific in vivo models to explore the anticancer properties.

Therapeutic studies of *M. zapota* must be interpreted with caution, as there is significant inter-study variability, and some of these studies have poorly designed experiments, as well as heterogeneous extraction procedures, dose levels, durations of treatment, and biological models. Differences in cultivar, plant part, solvent system, and analytical methods can cause the phytochemical composition and biological response to vary. To enhance the interpretation, this manuscript clearly distinguishes exploratory findings from evidence that has a stronger translational relevance and clearly discusses dose normalization, study limitations, and consistency of outcomes. The presentation of *M. zapota* extracts in the form of nanoparticles is not the same as conventional food applications and cannot be directly extrapolated to use in functional foods without targeted safety, bioavailability, and regulatory studies [57].

### 6.3. Anti-Inflammatory Activity

Inflammation, the immune response to injury, infections, and toxins, is triggered by pathogens, harmful foreign particles, allergic reactions, and autoimmune disorders. While acute inflammation has a protective effect, chronic and persistent inflammation leads to the development of complications like cardiovascular, metabolic, and autoimmune disorders. Inflammation modulates inflammatory cytokines, such as IL-6, TNF-α, IL-1β, IL-8, IL-10, NF-κB, CRP, LPO, and COX-2, consequently elevating the prevalence of inflammatory disorders. This has encouraged the exploration of medicinal plants for reducing such complicated ailments. Experimental studies have demonstrated that *M. zapota* possesses significant anti-inflammatory potential. Phytochemicals, such as flavonoids, tannins, and phenolics, are responsible for its anti-inflammatory activity [77,78]. Prenylated coumarins extracted from the fruit attenuated inflammation by suppressing lipopolysaccharide-induced nitric oxide (NO) production [79]. Furthermore, the anti-inflammatory activity of the methanolic bark extract (200 and 400 mg/kg) was investigated against carrageenan- and histamine-induced paw edema in rats. A significant reduction in paw edema was observed in the extract-treated groups [14,80]. Similarly, leaves (25–100 µg/mL) inhibited carrageenan-induced paw edema in rats. The ethyl acetate and ethanolic extracts (~300 mg/kg) reduced inflammation by 91.9% and 92.4%, respectively [16,81].

Another study evaluated the anti-inflammatory effect of leaves in aqueous, ethanolic, and hydroalcoholic extracts in 12-O-tetradecanoylphorbol-13-acetate-induced ear edema via a murine model. It has been shown that the ethanolic extract (5 mg) had more anti-inflammatory potential than the other extracts [58,82]. Similarly, aqueous leaf extract reduced ROS production and proinflammatory cytokines, such as TNF-α and IL-6, thereby improving colon dysplasia in BALB/C mice [83]. The antioxidant compounds in *M. zapota* play a crucial role in its anti-inflammatory and metabolic regulatory properties, as oxidative stress is closely associated with inflammatory signaling pathways. The majority of experimental in vivo and in vitro studies have shown the anti-inflammatory potential of *M. zapota*; however, the exact molecular pathways of these anti-inflammatory activities need further elucidation. Thus, future mechanistic and clinical studies are needed to establish the clinical significance in managing inflammatory diseases.

### 6.4. Cardioprotective Potential

Cardiovascular disorders (CVD_S_), such as hypertension, cardiac arrest, and myocardial infarction, are continuously increasing, contributing to an increased mortality rate worldwide. Dyslipidemia, oxidative stress, and chronic inflammation are the major contributors to the development of CVDs, which are caused by a high-fat diet, limited intake of fruits and vegetables, reduced physical activity, and medications [84]. Certain medications, i.e., statins, ezetimibe, PCSK9 inhibitors, and bile acid sequestrants, are available to improve the lipid profile, but their adverse effects highlight an urgent need for food-derived candidates, which require further validation [85,86]. Medicinal plants like *M. zapota* have revealed hypolipidemic effects by improving HDL-cholesterol levels and suppressing LDL-cholesterol, triglycerides (TG), total cholesterol (TC), and proinflammatory cytokines, ultimately modulating biomarkers associated with cardiovascular risk in experimental models [58,87]. The leaf extract has shown hypolipidemic activity in hyperlipidemic rats. Recent studies reported that treatment with leaves and fruits leads to significant reductions in cholesterol and triglycerides and increases HDL (high-density lipoprotein)-cholesterol levels [88].

**Figure 4:**
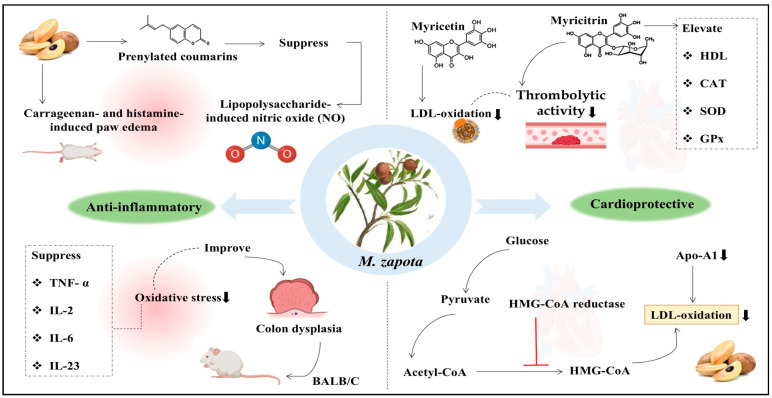
Anti-inflammatory and cardioprotective mechanisms of *M. zapota*. The bioactive constituents and their associated pharmacological actions, along with molecular mechanisms, are pictured. Prenylated coumarins exhibit anti-inflammatory activity by suppressing carrageenan- and histamine-induced paw edema and inhibiting lipopolysaccharide (LPS)-induced nitric oxide (NO) production. The extract further downregulates proinflammatory cytokines, including tumor necrosis factor-alpha (TNF-α), interleukin-2 (IL-2), interleukin-6 (IL-6), and interleukin-23 (IL-23), thereby reducing oxidative stress and improving colon dysplasia in BALB/c mice. Flavonoids such as myricetin and myricitrin contribute to cardioprotective effects by decreasing LDL oxidation and thrombolytic activity while enhancing antioxidant defense systems, including superoxide dismutase (SOD), catalase (CAT), and glutathione peroxidase (GPx). Moreover, modulation of glucose metabolism and inhibition of 3-hydroxy-3-methylglutaryl-coenzyme A (HMG-CoA) reductase reduce cholesterol biosynthesis and Apo-A1–mediated low density lipoprotein (LDL) oxidation.

The cardiovascular marker-related properties of *M. zapota* are associated with its antioxidant activity and enzymatic pathway regulation. Ethanolic leaf extract (100 and 300 mg/kg) significantly improved lipid profile, i.e., increased HDL-cholesterol levels and decreased LDL-cholesterol and Apo-A1. It is also observed that flavonoids prevented LDL-oxidation and cholesterol synthesis by inhibiting HMG-CoA reductase activity [89]. Furthermore, *M. zapota* leaves modulated cholesterol levels by suppressing pancreatic cholesterol esterase activity [89]. Angioprotective properties and proteasome activities were demonstrated in rabbits with cholesterol-induced atherosclerosis. Myricetin lowered LDL-oxidation and reduced the incidence of CVDs [90]. Furthermore, ref. [91] evaluated the antiatherosclerotic potential of myricitrin in hypercholesterolemic rats by treating them with myricitrin (100 µM) for 45 consecutive days. The results showed reduced levels of LDL-cholesterol, TC, TG, reactive oxygen species (ROS), and lipid peroxidation. Moreover, myricitrin elevated HDL-cholesterol, catalase (CAT), superoxide dismutase (SOD), and glutathione peroxidase (GPx) levels (Figure 4). The aortic wall surface area was also lowered by about 2%, 4%, and 27% by administration of 1, 10, and 100 µM myricitrin, respectively. Additionally, the in vitro thrombolytic activity of the hydroethanolic leaf extract was investigated. The results revealed that ~100 µg/mL extract attenuated blood clotting by about 23.9%, which was mainly due to the presence of phytoconstituents such as glycosides, tannins, terpenoids, and flavonoids [92]. Hence, the cardioprotective properties are largely substantiated by in vivo and in vitro experimental evidence, which has revealed improvements in lipid profiles and antioxidant defense mechanisms; more long-term in vivo clinical trials are encouraged to confirm this cardioprotective effect. The anti-inflammatory and cardioprotective mechanisms of *M. zapota* are summarized in Figure 4.

### 6.5. Hepatoprotective and Osteoprotective Potential

The liver is a vital organ of the body that is involved in metabolizing nutrients and drugs, synthesizing enzymes and hormones, producing bile, and detoxifying harmful chemicals and substances. These functions are disturbed by unhealthy eating patterns, a sedentary lifestyle, and exposure to environmental contaminants, which in turn trigger the propagation of hepatocellular disorders. Medicinal plants are gaining importance in healthcare sectors in supporting research on liver-related biomarkers [93]. *M. zapota*, one such medicinal plant, has shown liver marker-modulating effects in animal models, as evidenced by various studies. The hepatoprotective potential of 250 and 500 mg/kg fruit extract was evaluated in CCL_4_-intoxicated and hepatic-injured rats. The results revealed significant reductions in serum bilirubin and liver enzyme markers in treated animals, such as alanine aminotransferase, alkaline phosphatase, and aspartate transaminase. Moreover, free radical production and lipid peroxidation were also reduced [94,95]. Furthermore, it has been observed that the hepatoprotective potential is due to its phytoconstituents, such as polyphenols (methyl 4-O-galloylchlorogenate), which exhibit potent antioxidant attributes [18].

Similarly, bones are the fundamental elements of the skeleton, which grows rapidly during the second decade of life [96]. Environmental, genetic, dietary, and lifestyle factors directly or indirectly influence the development and breakdown of bones [97,98,99]. Studies have reported that high protein consumption modulates acid production and renal acid excretion, thereby regulating bone resorption and urinary calcium excretion [100,101]. Calcium modifications and circulating parathyroid hormone concentration also influence bone balance and the bone remodeling rate. Moreover, lower caloric intake is associated with reduced bone density and bone formation [102,103]. These factors ultimately weaken bone strength and aggravate the risk of osteoporosis and imbalanced stature. Multiple medications, such as calcium, phosphorus, and vitamin D supplements, are commonly used for strengthening bones, with variable success, but rural communities cannot afford these drugs and supplements due to inflation and high costs [104,105]. Therefore, scientists and healthcare professionals are trying to develop less expensive, natural, and effective substitutes to treat osteoporosis and other health disorders. Medicinal plants like *M. zapota* have shown antiarthritic-related activity in experimental models, which we will now describe in detail. An in vitro study was conducted using a protein denaturation model to evaluate the effect of *M. zapota* on rheumatoid arthritis (RA). Treatment with an ethanolic extract (100 and 250 μg/mL) can be used to reduce protein denaturation by 58.8% and 75.8%, respectively [13]. In another study, aqueous leaf extract (400 mg/kg) reduced paw edema, while gold nanoparticles had a relatively greater influence in sub-acute arthritis, with an inhibition rate of 83.4%. Moreover, enzymatic activity, such as alanine transaminase (ALT), aspartate transaminase (AST), and alkaline phosphatase (ALP), was also decreased under sub-acute arthritic conditions by the gold nanoparticles [38]. Although experimental rodent models and preclinical research have shown the hepatoprotective and osteoprotective potential, the clinical significance is still questionable. Therefore, further mechanistic and clinical human trials using standardized extracts are required to support the biological relevance and clinical applicability. There is preliminary evidence of hepatoprotection and osteoprotection, as most of the findings are from animal arthritis models, protein denaturation assays, and chemically induced liver injury models. Such models are valid for screening purposes but cannot be used to confirm clinical efficacy. There are also various studies using extracts or nanoparticles that are not comparable with the usual dietary consumption of sapodilla. These effects should thus be reported as observations from experiments and need to be standardized using extracts and be tested for dose–response effects and clinically validated.

### 6.6. Antidiabetic Activity

Diabetes mellitus (DM) is a rapidly progressive disorder characterized by the prolonged elevation of blood glucose levels due to inadequate insulin production or impaired body response to insulin. Approximately 25% of the world population is influenced by DM [106]. Sedentary lifestyle, genetic mutation, environmental variants, autoimmune disorders, and hormonal changes are involved in the pathogenesis of DM. Diabetes mellitus leads to the progression of hypertension, cancer, osteoporosis, stroke, hyperlipidemia, cardiac arrest, and gastroesophageal reflux disorders (GERDs). The elevated global prevalence of DM has intensified the development of food-derived bioactive compounds, specifically derived from medicinal plants, with minimal adverse consequences. Several studies have reported glucose-lowering effects in experimental models of *M. zapota* extracts using rodent modeling. Overall, these studies showed that extracts derived from the fruits, leaves, and seeds have glucose metabolism-related effects in preclinical models. The hypoglycemic effect is attributed to the bioactive compounds that are present [89,107]. Moreover, refs. [9,89] evaluated the hypoglycemic effect of fruit, pulp, and leaves using rat models. The extracts significantly reduced blood glucose, TC, and TG, lowering the incidence of weight gain and its associated health consequences. Moreover, a comparative study was conducted to evaluate the antidiabetic potential of the ethanolic and aqueous extract of seeds. The results showed that the ethanolic extract has more blood glucose-lowering potential than the aqueous seed extract [108].

Drugs such as alloxan, streptozotocin, nicotinamide + streptozotocin, and dexamethasone induce DM in rats. Ref. [109] conducted a study to investigate the hypoglycemic effect of leaves, fruit, and pulp in streptozotocin-induced diabetic rats. The leaf extract and pulp extract lowered glucose, TC, and TG levels. Similarly, the aqueous and alcoholic leaf and fruit extracts lowered glucose levels in alloxan-induced diabetic rats [110]. Moreover, the antidiabetic potential of *M. zapota* is largely associated with its ability to inhibit digestive enzymes, particularly α-amylase and α-glucosidase, that are involved in carbohydrate metabolism. *M. zapota* leaves have demonstrated their potential in suppressing the activity of α-amylase, which hydrolyzes complex polysaccharides into oligosaccharides and simple carbohydrates [111]. Furthermore, polyherbal tablets prepared from leaf extracts also exhibited hypoglycemic effect in alloxan- and streptozotocin-induced DM in rats [112,113,114]. Overall, the antidiabetic potential appears to involve multiple mechanisms, such as carbohydrate-digesting enzyme inhibition, antioxidant protection, and modulation of metabolic pathways. Further, the hypoglycemic potential is described in Table 3. Rodent and in vitro studies have demonstrated the hypoglycemic potential, in that it inhibits the activity of α-amylase and α-glucosidase and enhances lipid metabolism. However, differences in extraction techniques and dose regimens across studies make it difficult to interpret outcomes, as clinical trials are limited. Thus, effective clinical trials should be performed to evaluate the effectiveness, safety, and standard dosage of *M. zapota* for DM management. The antidiabetic evidence is based on analyses of carbohydrate-digesting enzymes and diabetic rodent models [115]. Some studies have shown α-amylase and α-glucosidase inhibition, while others have shown reduction of blood glucose and an improvement in the lipid profile in alloxan- or streptozotocin-induced models. However, the studies vary in their plant parts, solvent extracts, doses, models, and intervention durations. The differences make it difficult to find an effective dietary dose or standardized extract. In this case, *M. zapota* must be viewed as having possible glucose metabolism-supporting properties and not as being an antidiabetic treatment [116].

### 6.7. Gastroprotective Potential

Gastric health has a significant association with harmonizing the normal health of individuals, as the gastrointestinal tract is involved in food digestion, nutrient absorption, microbial balance, hormonal regulation, immune function, enzyme secretion, and waste elimination [117,118]. Any disturbance by drugs, autoimmune and genetic disorders, contaminants, poor dietary practices, and allergies leads to serious gastrointestinal complications, such as gastritis, gastroesophageal reflux disorders (GERDs), peptic ulcers, irritable bowel syndrome (IBS), foodborne infections, diverticulitis, small intestinal bacterial overgrowth (SIBO), and fatty liver disorders (FLDs) [119,120,121]. Increasing interest has been directed towards plant-based therapies due to their health promotion with limited adverse effects. Several studies revealed that *M. zapota* has digestive support-related effects in experimental models because of its phytoconstituents. Experimental studies have shown that extracts suppress gastrointestinal disturbances. Diarrhea is a serious gastrointestinal complication, leading to dehydration and micronutrient deficiencies [122]. It has been observed that *M. zapota* leaves have diarrhea-reducing properties, which is particularly attributed to the inhibition of prostaglandin (PG) biosynthesis in the intestinal mucosa. Moreover, they are also involved in the suppression of phosphodiesterase receptors and Ca^2+^ channels, ultimately inducing laxative effects [123]. Moreover, the antidiarrheal effect of ethanolic bark extract was evaluated in a castor oil-induced diarrhea model in rats by administering 200 and 400 mg/kg of bark extract. It has been found that the ethanol extracts (200 mg/kg and 400 mg/kg) notably reduced defecation by 53.5% and 60.7%, respectively, while loperamide reduced defecation by 71.43% [9]. These findings suggest that *M. zapota* improves digestive stability by regulating fluid secretions and intestinal contractions.

The gastroprotective effects are also associated with antiulcer activities, as studies have shown that extracts attenuate ulcer formation and gastric mucosal damage. For instance, ref. [124] conducted an in vitro, proteomic, and in silico study to evaluate the anti-motility and ulcer index-related effects in animal or in vitro models of aqueous and chloroform extracts of fruits. It has been observed that both extracts dose-dependently protected against ethanol-induced ulcer formation and gut hyperactivity. Furthermore, proteomic analysis revealed a significant reduction in IL-18 levels, and the in silico study showed similar results as those observed in the in vitro study. Later, the antidiarrheal potential of ethanolic bark extract (250 and 500 mg/kg) was evaluated. The results showed a reduction in fecal output, i.e., 29.31% and 41.37% by 250 mg/kg BW and 500 mg/kg BW dosage, respectively [13]. Another critical aspect of gastrointestinal health is microbial dysbiosis. It is obvious that bioactive compounds, such as catechins, help in regulating intestinal microbial homeostasis by promoting metabolite synthesis. It has been revealed that catechins act as prebiotics, which support the growth of beneficial bacteria and inhibit pathogenic bacteria, thereby improving gut health [125].

**Figure 5:**
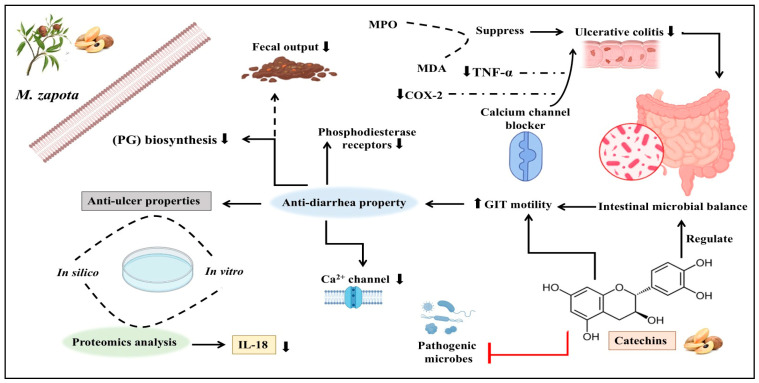
Mechanistic overview of the antidiarrheal and antiulcerative effects of *M. zapota*. The schematic diagram illustrates the multifaceted biological actions of *M. zapota*, highlighting its role in reducing fecal output through inhibition of PG biosynthesis and modulation of phosphodiesterase receptors. The antidiarrheal activity is mediated via suppression of Ca^2+^ channels, contributing to decreased gastrointestinal (GIT) motility. Additionally, catechins present in *M. zapota* regulate intestinal microbial balance by inhibiting pathogenic microbes. Anti-inflammatory effects are evidenced by the downregulation of myeloperoxidase (MPO), malondialdehyde (MDA), tumor necrosis factor-alpha (TNF-α), and cyclooxygenase-2 (COX-2), thereby alleviating ulcerative colitis. Proteomic analysis further supports reduced interleukin (IL-18) expression. These combined mechanisms contribute to its antiulcer and gut-protective properties, validated through in silico and in vitro approaches.

*M. zapota* fruit is effective against gastrointestinal disorders, such as gastritis, constipation, IBD, and diarrhea. The in vitro and in silico effects on gastric disorders were assessed. For instance, ref. [126] investigated the effect of ethanolic bark extract in managing ulcerative colitis (UC). The results revealed its potential in alleviating the severity of UC by suppressing myeloperoxidase (MPO) activity and colonic malondialdehyde (MDA) levels. Moreover, the gastroprotective property of methanol bark extract was assessed in Wistar rat pylorus ligation–, indomethacin-, and ethanol-induced ulcer models. The results showed that ~300 mg/kg of methanol extract may alleviate free acidity, the ulcer index, and gastric volume as part of its antiulcer characteristics [127]. Furthermore, leaf extracts are used to formulate a mouth ulcer gel formulation, which was evaluated for ulcer-related outcomes, and it has been found that it could alleviate ulcers within the body, specifically mouth ulcers [128]. Another study demonstrated that reduced gastrointestinal tract (GIT) motility increases the retention of substances in the small intestine, thereby enhancing water absorption and intestinal functionality. Moreover, the calcium channel blocker action of *M. zapota* suggested possible activity in the tested model in reducing the progression and severity of ulcers. Additionally, the antioxidant effect and nitric oxide (NO) free radical scavenging capability further modulates antiulcer activity by ameliorating oxidative stress in GI tissue. Similarly, rats treated with fruit extract showed comparatively lower TNF-α and COX-2 expression levels compared to those receiving omeprazole treatment, demonstrating the anti-inflammatory profile of the extract. Likewise, the administration of 5 g/kg *M. zapota* did not cause any toxicity or mortality in treated rats [124]. Figure 5 depicts the mechanism of events that promote gut health. Numerous in vivo and in vitro studies have demonstrated the gastroprotective effects via antioxidant and anti-inflammatory activities. However, the direct potential effect on human gastrointestinal disorders is still ambiguous; as a result, additional clinical studies and dosage testing should be conducted to confirm these effects. The gastroprotective evidence includes antidiarrheal, antiulcer, and gut motility outcomes, particularly in animals and in vitro studies. However, these models have different endpoints, such as fecal output, ulcer index, intestinal motility, inflammatory mediators, and mucosal protection. Extract types and doses are also inconsistent. Therefore, these findings suggest possible digestive support properties, but they do not justify clinical claims for treating diarrhea, ulcers, and other gastrointestinal diseases.

### 6.8. Antiaging Mechanisms

Aging is a complex and integrated process characterized by a time-dependent decline in functional ability and quality of life [129]. Modernization and industrialization have advanced early aging and reduced the life span. As a result, maladies such as cancer and cardiovascular, musculoskeletal, immune system, and neurodegenerative disorders are rapidly prevailing among communities [130]. Sedentary lifestyles and poor eating patterns trigger the aging process by producing free radicals, reducing antioxidants, disrupting mitochondrial performance, and promoting minimal caloric consumption [131]. Moreover, epigenetic modifications such as RNA regulation, RNA modification, DNA methylation, and chromatin remodeling further aggravate the aging process [132]. Healthcare sectors are also developing strategies to promote healthy aging with advancement and digitalization [133]. Herbal plants and their compounds, such as *M. zapota*, have been explored to find their association with ameliorating or delaying the aging process among individuals. *M. zapota* has delayed aging owing to its free radical-scavenging potential, and relevant studies are explained here comprehensively. It has been found that extrinsic and intrinsic factors, like ultraviolet (UV) exposure and time, have a significant impact on skin aging, as they stimulate oxidative damage, ROS production, and metalloproteinase (elastase and collagenase) activity [134]. Depletion of collagen and elastin, essential proteins for preserving skin structure, promotes wrinkles and other aging problems, mostly linked to cognitive performance.

A comparative study performed to investigate the antiaging potential of *M. zapota* by analyzing its antielastase property, anticollagenase activity (of MMP-1 and MMP-2), and in vitro antioxidant capacity was conducted, which revealed strong inhibitory action against MMP-1, MMP-2, and elastases with IC_50_ values of 89.6, 86.5, and 35.7 mg/mL, respectively [135]. Similar results were observed by the administration of 60% ethanolic and 95% ethanolic fresh pulp extract at a 140 µg/mL concentration, which suppressed collagenase and elastase activities by about 66.4% and 64.7%, respectively [136]. Furthermore, a natural emulgel sunscreen was prepared using fruit extract to evaluate its free radical-scavenging potential under UV radiation conditions. The formulation was reported as nonirritating, as well as exhibiting UV-related protective activity. Moreover, leaves demonstrated a potent elastase-inhibitory effect compared to fruits [137]. Table 4 describes the overall pharmaceutical and nutraceutical attributes of different parts and extracts of *M. zapota*. Briefly, studies have implicated the antioxidant properties and anticollagenase/antielastase activities of *M. zapota* in its anti-aging effects. Nevertheless, the existing literature is mostly based on in vitro and in vivo studies, and the lack of clinical studies limits the ability to clarify the biological relevance and consumer/product applicability of *M. zapota* in modulating aging-related enzyme markers. Future research will involve examining the bioavailability, safety, and long-term effectiveness of *M. zapota* in dermatological and antiaging processes. Only enzyme inhibition, antioxidant activity, and some formulation-based studies have been used to support the antiaging property. The data are useful for product development, cosmetics, and functional ingredients, but do not support antiaging properties in humans. Long-term safety, penetration of the skin, formulation stability, and clinical acceptability should be studied further.

### 6.9. Integrated Mechanistic Insights

The therapeutic potential of *M. zapota* is particularly linked to various overlapping molecular pathways. Its bioactive compounds, especially flavonoids, phenolic acids, and triterpenoids, serve as antioxidants by scavenging reactive oxygen species (ROS) and reactive nitrogen species (RNS) and enhancing endogenous antioxidant enzymes, such as superoxide dismutase (SOD), catalase (CAT), glutathione (GSH), glutathione peroxidase (GPx), and inducible nitric acid synthase (iNOS). Subsequently, the decrease in oxidative stress inhibits proinflammatory signaling pathways, such as NF-Kβ, IL-6, TNF-α, and COX-2, thereby suppressing prolonged inflammation. As oxidative stress and inflammation are the common factors in the pathogenesis of metabolic and degenerative disorders, the regulation of these processes promotes anticancer, antidiabetic, cardioprotective, and gastroprotective properties. Moreover, phytochemicals, including myricetin, kaempferol, and caffeic acid, modulate caspase-3 and -8, Wnt/β-catenin, and GSK-3β signaling pathways and inhibit tumor growth. Furthermore, the suppression of metabolic enzymes, such as alpha-amylase, alpha-glucosidase, and HMG-CoA reductase, is equally important in enhancing glucose and lipid metabolism. Collectively, these processes emphasize that the therapeutic potential of *M. zapota* is mediated by an integrated network of antioxidant defense, anti-inflammatory regulation, apoptosis induction, and modulation of enzymes involved in metabolism. Consequently, the pharmacological potential cannot be considered as independent, but rather interdependent on the biological actions of oxidative stress, inflammatory signaling, and modulation of cellular apoptosis pathways.

## 7. Safety and Toxicity

The safety of plants is a significant concern for consumers and healthcare professionals when using them regularly. Local inhabitants and traditional healthcare professionals have limited knowledge about safe and toxic doses, leading to unintentional accidental deaths in the past [142]. National and international regulatory authorities, such as the World Health Organization (WHO), the International Council for Harmonization of Technical Requirements for Pharmaceuticals for Human Use (ICH), the Food and Drug Administration (FDA), the National Institutes of Health (NIH), the European Medicines Agency (EMA), the European Food Safety Authority (EFSA), the Medicines and Healthcare products Regulatory Agency (MHRA), the Natural and Non-prescription Health Products Directorate (NNHPD), the National Medical Products Administration (NMPA), the Pharmaceuticals and Medical Devices Agency (PMDA), and the Drug Regulatory Authority of Pakistan (DRAP), are working to declare the safety and toxicity of a particular plant [143,144,145]. Moreover, in vitro, in vivo, and clinical trials are conducted to evaluate the effective dosage of plants for facilitating their therapeutic attributes. Several experimental studies have involved acute toxicity trials in animal models and demonstrated the toxicity of *M. zapota*. Acute toxicity studies have reported that extracts are safe within a broad dosage range. For instance, ref. [14] evaluated the acute toxicity of methanol and chloroform leaf extracts in mice. The results revealed that the extracts did not cause any toxicity or mortality up to a dose of 2000 mg/kg body weight, demonstrating the lethal dose (LD_50_) for both extracts to be above 2000 mg/kg body weight. Similarly, the ethanolic fruit extract was orally administered to rats at 200, 400, 800, 1600, and 3200 mg/kg body weight to evaluate its toxic effect on rat health. No significant changes were observed in behavior, respiration, skin effects, sensory nervous system responses, and gastrointestinal functions during the observation period, demonstrating that the lethal dose is higher than 3200 mg/kg body weight (BW) under the experimental conditions [16]. These studies indicated that extracts are considerably safe when administered at a controlled and optimum dosage. However, it has been observed that the toxicity data were evaluated only in short-term acute toxicity studies; therefore, long-term studies of toxicity, pharmacokinetics, and clinical safety in humans should be conducted to validate the safety of *M. zapota* for pharmaceutical and nutraceutical applications. Another limitation of the existing evidence is that there are no comprehensive pharmacokinetic and metabolic investigations on the absorption, distribution, metabolism, and excretion of the phytochemicals; without these, predicting possible bioaccumulation, metabolic interactions, or long-term toxicity risks of routine ingestion of concentrated extracts is difficult to determine. The existing safety data for *M. zapota* are not comprehensive or fully explored. Ripe fruit pulp is traditionally used for nutrition; but extracts, seeds, bark, leaves, latex, peel concentrates, and nanoparticle formulations need a separate safety assessment. Moreover, high-dose acute animal studies indicating no mortality cannot prove chronic safety, NOAEL, LOAEL, ADI, reproductive safety, genotoxic safety, or human tolerability. Furthermore, cyanogenic glycosides have been reported, and exposure to cyanide should be measured in raw and processed products and compared to established toxicological reference values. Future work should be conducted according to OECD repeated dose toxicity guidelines, EFSA botanical safety guidelines, and FDA guidelines for the safety of foods as ingredients. Until this type of evidence is available, only precautionary statements regarding traditional use of fruits and initial acute toxicity data should be made. *M. zapota* is underutilized compared with other fruits, but its potential in the food industry is notable. *M. zapota* has significant potential as a source of value-added food products; however, its industrial application should be defined in terms of shelf life, processing conditions, quality indicators, sensory acceptability, and commercialization.

## 8. Applications in Food Systems

*M. zapota* is underutilized compared to other fruits; however, its potential in the food industry is considerable. It shows significant promise as a source of value-added food products, although its industrial applications must be evaluated in terms of shelf life, processing conditions, quality indicators, sensory acceptability, and commercialization potential. Fresh fruits are highly perishable, with a shelf life of 6–9 days under optimal storage conditions. Storage at 12–16 °C and 85–90% relative humidity (RH) can extend shelf life up to 3 weeks, whereas storage beyond 10 days at 4 °C may lead to chilling injury. Therefore, post-harvest losses can be minimized, and industrial utilization can be enhanced by processing the fruit into jams, spreads, fruit bars, slices, pastilles, juices, and ready-to-serve (RTS) beverages [18]. Different parts of the plant, including fruits, seeds, and leaves are utilized in the beverage, dairy, confectionery, baking, and cosmetic industries.

### 8.1. Confectionery Industry

The high carbohydrate content (14–20%), sugars (11–15%), and reducing sugars (7–9%) in the fruit make it a strong candidate for the confectionery industry. Fruit pulp and pectin have been used to prepare antimicrobial jams, where high-pressure processing (300–600 MPa) for 0–30 min at 25–65 °C aids in peroxidase inactivation. Furthermore, the enzyme inactivation rate was more sensitive to temperature and pressure under combined high-pressure and high-temperature conditions [21]. Optimization studies on spreads formulated using pulp, grape juice, pectin, and citric acid have also been conducted. The optimized formulation consisted of an *M. zapota*-to-grape ratio of 1.14, 0.58 g/100 g pectin, 0.21 g/100 g citric acid, 29 °Brix total soluble solids (TSSs), 65% moisture, 11.44 mg GAE/g phenolics, antioxidant capacity of 83.2 mg GAE/kg, and firmness of 47.3 N. The formulation with the highest sensory acceptability was identified using fuzzy logic analysis [146]. However, moisture, firmness, pH, sugar content, and sensory balance require careful control during scale-up to support the development of reduced-calorie and antioxidant-rich sapodilla spreads.

Dried *M. zapota* products such as fruit bars and refractance window-dried bars are promising due to their reduced water activity and improved portability. The hardness, springiness, cohesiveness, adhesiveness, and chewiness of pectin-based fruit bars improved with reduced moisture content and water activity. The optimized refractance window drying conditions were 91 °C drying temperature, 5 mm pulp thickness, 2% pectin, and 146 min drying time, resulting in bars containing approximately 16 g H_2_O/100 g moisture and 10.7 mg/100 g ascorbic acid [147]. Refractance window drying can reduce drying time, temperature, cost, energy consumption, and nutrient loss while preserving color, aroma, antioxidant compounds, and overall nutritional quality. Nevertheless, further research is required to scale up the technology for commercial applications.

Reduced-calorie, high pressure-processed jam was developed using sapodilla pulp containing 45% TSS, 4.5% pectin, and 0.5% citric acid. The jam demonstrated desirable rehological, textural, sensory, and storage properties after processing at 400 MPa for 10 min at 27 ± 1.5 °C [21]. These findings suggest that sapodilla jam has potential as a reduced-calorie functional food product; however, challenges related to enzyme inactivation, equipment cost, process validation, and product positioning remain to be addressed. Another value-added confectionery product developed from *M. zapota* is pastilles. Six purée formulations (10%, 15%, 20%, 25%, 30%, and 40%) were evaluated. The 12% purée formulation showed the highest overall sensory acceptability, with 62.7 °Brix, pH 4.3, and water activity of 0.54, whereas the 20% purée formulation exhibited higher moisture, ash, and fiber contents and higher antioxidant activity. The product retained desirable texture and color after 2 months of chilled storage, indicating commercial potential without the use of synthetic preservatives or artificial coloring agents [148]. In another study, pectin extracted from sapodilla peel was used to prepare pudding with superior quality compared to puddings prepared using pectin extracted from banana and mango peels. Puddings prepared with extracted and commercial pectin showed similar characteristics and no significant differences in texture [149].

### 8.2. Beverage Industry

The highest hedonic acceptability for formulated juice was achieved with 50% pure juice, 25 °Brix, and 0.40% titratable acidity. The formulated juice had a pH of 3.35 and exhibited superior microbial stability compared to pure juice. No microbial colony formation was observed after 1 week of storage at 28 °C, whereas pure juice showed 169,318.18 CFU/mL after the same storage period [150]. In addition, sonicated and microwave-processed RTS beverages were evaluated over 90 days of storage. The treated beverages exhibited superior color, flavor, taste, and overall sensory attributes, while also retaining higher flavonoid and antioxidant contents than untreated controls [151]. The findings suggest that industrial-scale production of *M. zapota* beverages requires acidification, mild/non-thermal processing, hygienic filling, and proper storage validation.

*M. zapota* bars and juices prepared from fresh fruit demonstrated strong antioxidant activity (DPPH and FRAP assays), total phenolic content (TPC), total flavonoid content (TFC), and tannin content in 70% acetone extracts compared to water and ethanol extracts. Moreover, the 70% acetone extracts exhibited strong antibacterial activity against *Escherichia coli*, *Staphylococcus aureus*, *Bacillus subtilis,* and *Salmonella* spp. [152]. Furthermore, the addition of maltodextrin and gum arabic has been shown to preserve juice quality, antioxidants, and phytochemicals by preventing oxidation [153]. Probiotic *M. zapota* juice was prepared using *Lactobacillus plantarum, Lactobacillus bulgaricus, Lactobacillus acidophilus,* and *Lactobacillus casei,* followed by incubation at 30 °C and 37 °C for 3 days. The resulting product exhibited lower pH and titratable acidity, increased TPC, and no significant changes in sensory attributes [154].

### 8.3. Dairy Industry

The dairy industry plays an important role in maintaining health by providing proteins, carbohydrates, fats, vitamins, and minerals. Daily consumption of two to three servings of dairy products is generally recommended to meet nutritional requirements. Drinking yogurt has been developed using different proportions of fruit powders, including *M. zapota, Cynometra cauliflora*, *Flacourtia indica*, and *Elaeocarpus serratus.* The addition of these fruit powders increased titratable acidity, reduced pH, improved nutritional composition, and extended shelf life by reducing yeast, mold, coliform and total plate counts [155]. Interestingly, incorporation of 5% ripened *M. zapota* pulp into milk yogurt significantly reduced pH (4.0–4.2), improved protein content (5.58–5.73%) and total solids (29.34–29.64%), enhanced consumer acceptability, and reduced product cost (*p* < 0.05) during 15 days of refrigerated storage [156]. Due to the perishable nature of *M. zapota*, food technologists have also developed RTS beverages using processing techniques such as sonication and microwave treatment. The physiochemical characteristics, including acidity, pH, TSS, antioxidant activity, and sensory attributes, were evaluated. The results indicated no significant adverse effects on nutrients, phytochemicals, juice color, or bioactive compounds [151].

### 8.4. Tea Industry

Tea is widely consumed beverage and an important source of bioactive compounds that are beneficial against various diseases. Infused dried *M. zapota* powder has been incorporated into different milk teas, including fat-free cow milk, soy milk, almond milk, and lactose-free cow milk. These milk teas exhibited lower glycemic indices, retained antioxidant activity, and achieved higher consumer acceptability scores (7.6) compared to commercial milk tea [157]. Similarly, herbal tea prepared using *M. zapota* fruit powder was evaluated using an electronic tongue (e-tongue) system for sensory attributes such as aftertaste, astringency, sourness, and bitterness. The herbal tea demonstrated good quality and consumer acceptance [158].

### 8.5. Baking Industry

The bakery industry plays an important role in the food sector and serves as a medium for fortification of products with minerals, vitamins, dietary fiber, proteins, carbohydrates, fats, and bioactive compounds. It produces bread, cakes, pastries, and cookies that serve as staple foods in many diets worldwide. Cookies prepared with sapota fiber powder (7%) and beetroot leaf powder (4.5%) were evaluated for storage stability over 15 months. Cookies containing 7% sapota fiber powder maintained lower moisture content, peroxide value, free fatty acids, and microbial growth while exhibiting superior preservation compared to reference cookies. Moreover, sapota fiber powder cookies demonstrated greater consumer acceptability than beetroot leaf powder cookies, indicating their potential for enhanced shelf life and quality [159]. Fiber-rich biscuits incorporating dehydrated chiku powder and dry fruit fillings (figs and dates) have also been developed as dietetic bakery products with enhanced nutritional value. Figs contributed higher ash and protein contents, whereas dates provided dietary fiber along with significant amounts of chlorogenic acid and protocatechuic acid. Incorporation of chiku powder reduced dough properties such as water absorption and extensibility; however, biscuits with higher °Brix fruit fillings exhibited improved baking stability and breaking strength. The use of sodium alginate further stabilized the fillings and enhanced their sensory and functional properties, while increasing dietary fiber and phenolic acid content [160]. Fortified buns with varying levels of pearl millet flour (PMF) and sapota powder were evaluated for shelf life and sensory attributes over 6 days. The optimized formulation containing 22% pearl millet flour and 12% sapota powder received the highest acceptability scores on a nine-point hedonic scale. However, by the sixth day of storage, reductions in carbohydrate, protein, ash, energy, and total flavonoid contents were observed, along with the growth of yeast and molds in both optimized and control buns [161]. Therefore, further research is needed to optimize processing conditions and dosage levels to improve the utilization of *M. zapota* in the baking industry.

## 9. Cosmetic Industry

The cosmetic industry is growing day by day. The use of bio-green sources is gaining attention to make effective cosmetic products. Hydro-alcoholic extract of *M. zapota* as a sunscreen was evaluated and did not undergo any photodegradation when exposed to UV, and the growth of *Candida albicans* was not affected. The spectral analysis revealed no considerable change in absorption between 250–320 nm, while the linoleic acid peroxidation test indicated negligible peroxide formation, suggesting higher photostability of the extract than the standard sunscreens [162].

The emulgel containing *M. zapota* fruit extract (MZFE) was tested to assess the efficacy of this extract in terms of sun protective factor (SPF) and physical retention on the facial skin. The formulation proved to be most stable physically and chemically and also demonstrated good in vitro (14.215 ± 0.140) and in vivo (SPF 14.215 ± 0.3) photoprotective properties, which lasted for about 120 min. The MZFE-loaded emulgel formulation proved to be effective in providing UV filtering properties, and hence it could be used as a possible material in sun protection products [137]. The effects of sapodilla leaf extract cream on PDGF and IL-10 levels were tested in UVB-exposed Wistar rats. The group treated with the 50% sapodilla leaf extract cream showed the highest level of PDGF, and the difference was found to be significant among the various treatment groups (*p* = 0.024). The levels of IL-10 were similar in both groups (*p* = 0.240), indicating that the cream could have a greater impact on the production of PDGF, but not on the production of IL-10. The results indicate that sapodilla leaf extract has the capability to heal UVB-induced skin damage [163].

## 10. Pharmaceutical Industry

*M. zapota* has numerous pharmaceutical and nutraceutical applications due to its wide range of phytochemicals, such as flavonoids, phenolic acids, triterpenoids, and glycosides. These constituents are responsible for their antioxidant, anti-inflammatory, anticancer, and antidiabetic potential, making them an ideal source of natural therapeutic agents in the pharmaceutical industry. Moreover, it can also be used in novel drug delivery systems, as recent studies have used its extracts in metal nanoparticle synthesis. Furthermore, it can be used in the preparation of functional foods, dietary supplements, and nutraceuticals due to its nutrient and bioactive compound bioavailability. However, international regulatory agencies like the WHO, FDA, and EFSA require comprehensive toxicological and quality assessments prior to the incorporation of plant-based ingredients in the development of commercial products. An edible gel produced from M. *zapota* fruit pulp and the probiotic bacterium *Lactobacillus fermentum* A15 was developed by microencapsulation using a spray-drying process. The bioactive compounds and viability of the probiotic (1 × 10^7^ CFU/mL) were preserved by using this method, positioning it as a promising product for lactose-intolerant consumers and catering to the growing functional food market [164].

## 11. Limitations and Future Perspectives

In food applications, additional efforts are needed to prove industrial shelf life, storage stability, enzyme browning control, cost, packaging, consumer acceptance, and commercial scale-up. The antioxidant activity level of evidence is moderate to low. The free radical-scavenging activity has been reported by various studies with DPPH, ABTS, FRAP, and H_2_O_2_ scavenging assays; however, these assays primarily measure antioxidant activity, but cannot necessarily verify the biological antioxidant activity in humans. Furthermore, the antioxidant properties are difficult to compare, as the plant parts used for the extraction, solvents, concentrations used, standards used, and assay conditions are diverse.

In conclusion, the major industrial constraints for *M. zapota* products are the short shelf life of the fresh fruits, bruising, enzymatic browning, handling problems with the latex, seasonal availability, absence of standardized maturity indexes, lack of consumer awareness outside of tropical markets, and high cost of advanced processing technologies. It has been reported that maturity grading, gentle handling, pre-cooling, pulp freezing, acidification, pectin optimization, water activity control, sensory-led formulation, and staged commercialization can be used to reduce these challenges. Fruit bars, pastilles, dehydrated fruit slices, spreads, and beverages could be lower cost products that could be commercialized sooner, whereas HPP-based jams and higher-value refrigerated products might be more suitable for higher-value markets.

Anti-inflammatory, antidiabetic, gastroprotective, cardioprotective, hepatoprotective, osteoprotective, anticancer, and antiaging activities are also low to moderate, depending upon the endpoint. The majority of findings are from in vitro, cell culture, in silico, and animal models, which provide interesting mechanistic insights, including proposed modulation of oxidative stress, inflammatory cytokines, carbohydrate-digesting enzymes, lipid metabolism, markers of apoptosis, and enzyme inhibition. However, there is insufficient evidence to support the therapeutic efficacy in humans. Moreover, a number of extracts and isolated substances derived from *M. zapota* have been reported to have cytotoxic properties in cancer cell lines; however, cell line inhibition cannot be directly equated with clinical anticancer activity. There are variations in extract types, dosages, cell lines, exposure times, and mechanistic endpoints across the available studies. Furthermore, the selectivity, bioavailability, pharmacokinetics, safe dosage, and efficacy have not been well established. Thus, anticancer properties can only be reported as initial laboratory results.

Likewise, the antidiabetic and cardioprotective results are encouraging but inconclusive. Some studies reported that α-amylase and α-glucosidase inhibition was observed along with decreased blood glucose levels, a better lipid profile, and a decrease in oxidative stress markers. Comparisons are difficult due to variations in the diabetic induction models, preparation of the extracts, doses administered, durations of treatment, and measured endpoints. Clinical trials involving humans are required to confirm the use of extracts in diabetes and cardiovascular management.

Careful and cautious statements should also be made about safety. The available safety data for the extracts are limited, but some studies indicate that they are not particularly acutely toxic at the tested doses. Safety in vulnerable populations, herb–drug interactions, genotoxicity, reproductive toxicity, chronic toxicity, and organ-specific toxicity are not fully explored. Thus, there is insufficient evidence to guarantee complete safety of therapeutic or high-dose nutraceutical use.

## 12. Conclusions

*M. zapota* is a tropical fruit, rich in nutrients, containing a wide range of phytochemicals, such as phenolics, flavonoids, tannins, terpenoids, sterols, and alkaloids, which have been reported to have antioxidant, anti-inflammatory, metabolic, gastroprotective, cardioprotective, hepatoprotective, antiaging, and industrial attributes. However, there is varying evidence supporting these properties; therefore, assessment of the biological activity should be carried out to validate its effectiveness for clinical use. The available literature suggest that the nutritional composition and development of food products are moderate, since some nutritional studies have been performed to assess the proximate composition, phytochemical content, product formulation, physicochemical quality, and sensory acceptability of the products. In conclusion, *M. zapota* is a good source of nutrients, phytochemicals, and value-added food ingredients. It has so far the most promising practical applications in the food sector (jams, spreads, fruit bars, pastilles, dehydrated foods, and beverages). It has biological plausibility and is largely in the preclinical stages. Standardizing extract preparation, active compound identification, dose response, bioavailability, pharmacokinetic, chronic toxicity, and well-designed human clinical trials should be considered as future research areas.

## Figures and Tables

**Table 1:** Nutritional composition of, antioxidant activity of, and major bioactive compounds identified in different parts of *M. zapota*.

Plant Part	Nutritional Composition	Antioxidant/Phytochemical Potential	Major Bioactive Compounds	References
Fruits	Carbohydrates (14–20%), sugars (11–15%), proteins (0.52–0.76%), fats (0.6–1.1%), moisture (60–69%), dietary fiber, vitamins A and C, potassium, calcium, phosphorus, magnesium, iron	TPC: 20–85 mg GAE/g; TFC: 34.9 mg QE/g; DPPH: 61.3%; FRAP: 540 µmol Fe^2+^/g; ABTS: 65.2%	Gallic acid, chlorogenic acid, quercetin, catechin, epicatechin, myricetin, kaempferol, terpenoids, phenolic acids, flavonoids	[18,21,22,23,24,28,29,30,31]
Seeds	Oil (18–20%), oleic acid (40–50%), proteins, carbohydrates, dietary fiber, potassium, calcium, magnesium, phosphorus	TPC: 212.4 mg GAE/g; TFC: 97.6 mg QE/g; DPPH: 89.5%; FRAP: 1385 µmol Fe^2+^/g; ABTS: 91.2%	Gallic acid, epigallocatechin, catechin, vanillic acid, ethyl gallate, epigallocatechin gallate, β-carotene, flavonoids	[25,27,29,33]
Leaves	Unsaturated fatty acids, oleic acid, linolenic acid, vitamin C, potassium, calcium, magnesium, iron	TPC: 194.04 mg/g; TFC: 35.53 mg/g; DPPH: 93.8%; FRAP: 1640 µmol Fe^2+^/g; ABTS: 95.4%	Kaempferol, caffeic acid, quinic acid, β-sitosterol, stigmasterol, flavonoids, phenolic acids, triterpenes, sphingolipids	[25,26,27,28,29]
Pulp/Juice	Carbohydrates (19.9%), natural sugars (14.7%), dietary fiber (5.3%), vitamin C, vitamin E	TPC: 72.5 mg GAE/g; TFC: 28.6 mg QE/g; DPPH: 52.7%; FRAP: 410 µmol Fe^2+^/g; ABTS: 58.9%	Phenolic compounds, flavonoids, vitamin C, natural sugars	[18,24,36]

Abbreviations: TPC, total phenolic content; TFC, total flavonoid content; DPPH, 2,2-diphenyl-1-picrylhydrazyl; FRAP, ferric reducing antioxidant power; ABTS, 2,2′-azino-bis(3-ethylbenzothiazoline-6-sulfonic acid); GAE, gallic acid equivalent; QE, quercetin equivalent.

**Table 2:** Compounds isolated from *M. zapota* and their pharmacological activities.

Parts	Bioactive Compounds	Pharmacological Activities	Experimental Model	Findings	References
Fruit	L-arabinose	Antidiabetic	In vitro and in vivo	↓ Carbohydrate digestive enzymes, ↑glucose metabolism	[42]
	Dihydromyricetin	Antioxidant, Anti-inflammatory	In vitro and cell-based	↓ ROS (reactive oxygen species) production and inflammatory markers	[43]
	Methyl 4-O-galloylchlorogenat	Antiulcer, Antidiarrheal, Antioxidant	In vitro and in vivo	Protects gastric mucosa, ↓ ulcer formation	[44,45]
	Leucodephinidine	Antidiabetic, Antibacterial	In vitro	↑ Antimicrobial activity, glucose regulatory activity	[46,47]
	3-O-acetyl-D-methyl galacturonate	Antiviral	In vitro	↓ Viral replication	[42]
	4-O-galloylchlorogenic acid	Antioxidant	In vitro	↑ ROS scavenging	[44,45]
Leaves	D-quercitol	Antioxidant, Anti-inflammatory	In vitro	↓ Oxidative stress and inflammatory response	[48]
	Caffeic acid	Antioxidant, Antihepatocarcinoma, Anti-inflammatory	Cell culture and in vitro	↓ Oxidative damage and cancer cell proliferation	[49]
	Myricetin-3-O-alpha-L-rhamnoside	Antioxidant, Anti-inflammatory	In vitro	↑ Free radical scavenging potential and inflammatory response	[50]
Bark	Betulinic acid	Anti-HIV, Anticancer	In vitro	↓ Viral replication, ↑ apoptosis	[11]
	Oleanolic acid	Anticancer, Antidiabetic	In vitro and in vivo	↑ Glucose metabolism, ↓ cancer cell growth	[11]
	6-hydroxyflavanone	Antioxidant, Anti-Inflammatory, Anticancer	In vitro	↓ Oxidative stress, ↑ cytotoxic activity	[51]
	(+)- dihydrokaempferol	Anticancer, Antioxidant, Anti-hepatic fibrosis	In vitro and in vivo	↓ Oxidative stress and fibrotic pathways	[52]
	3,4-dihydroxybenzoic acid	Nematicidal, Antioxidant	In vitro	↑ ROS scavenging and nematicidal activity	[26]

ROS, Reactive oxygen species; HIV, Human immunodeficiency virus; *↑*, Increased; ↓, Decreased.

**Table 3:** Reported biological activities and proposed mechanisms associated with different parts of *M. zapota*.

Plant Part/Extract	Reported Biological Activity	Proposed Mechanism/Key Findings	Experimental Model	References
Fruit extract	Antioxidant activity	Reduced oxidative stress and scavenged free radicals due to phenolic and flavonoid compounds	In vitro assays	[42,43,44,45]
Fruit extract	Anti-inflammatory activity	Modulated inflammatory mediators and reduced nitric oxide production	In vitro/animal studies	[46,47,48]
Fruit extract	Antimicrobial activity	Inhibited the growth of selected Gram-positive and Gram-negative bacteria and fungi	In vitro studies	[49,50,51]
Fruit extract	Gastroprotective activity	Reduced gastric lesions and oxidative damage in experimental ulcer models	Animal studies	[52,53]
Fruit extract	Glucose metabolism-regulating activity	Improved glucose utilization and modulated carbohydrate-metabolizing enzymes	In vitro/animal studies	[54,55,56]
Leaf extract	Antioxidant activity	Increased antioxidant enzyme activity and reduced lipid peroxidation	In vitro/animal studies	[57,58,59]
Leaf extract	Anti-inflammatory activity	Reduced inflammatory cytokines and oxidative stress markers	Animal studies	[60,61,62]
Leaf extract	Antiproliferative activity	Induced apoptosis and inhibited proliferation in experimental cell line studies	In vitro studies	[63,64]
Seed extract	Antioxidant activity	High phenolic and flavonoid content contributed to radical scavenging activity	In vitro assays	[65,66]
Seed extract	Antimicrobial activity	Demonstrated inhibitory effects against selected microbial strains	In vitro studies	[67,68]
Bark extract	Antipyretic and analgesic activity	Reduced experimentally induced fever and pain responses	Animal studies	[69,70]
Bark extract	Traditional medicinal relevance	Traditionally used for astringent and febrifuge properties	Ethnobotanical reports	[9,10,11,12]

Abbreviations: in vitro, experimental studies performed outside living organisms; cytokines, inflammatory signaling proteins; oxidative stress markers, indicators associated with reactive oxygen species and cellular oxidative damage.

**Table 4:** Pharmacological characteristics of various parts (leaves, fruits, seeds, and bark) of *M. zapota*.

Biological Activity	Source	Solvent	Models	Mechanism of Action	Reference
Antioxidant activity	Leaves	Ethanol	In vitro	DPPH = 72.86 ± 11.22 μg/mL with IC_50_ = 7.92 ± 1.41 μg/mL	[49]
	Leaves	Methanol	In vitro	DPPH = 55.6 ± 1.6 μg/mL with EC_50_ = 30.6 ± 0.9 μg/mL	[138]
	Leaves	Water	In vitro	IC_50_ = 6.41 μg/mL	[139]
	Leaf, seed pulp, peel	Ethanol and water	In vitro	Aqueous and ethanol extract showed DPPH (%) values of 91.95 ± 1.21% and 78.23 ± 0.03%, respectively, for pulp; 82.02 ± 4.82% and 91.96 ± 0.73% for peel; 93.60 ± 0.56% and 92.93 ± 0.07% for leaves; 62.96 ± 0.21% and 49.35 ± 0.91% for seeds	[25]
	Fruit peel	n-Hexane, chloroform, acetone, ethanol, and water	In vitro	DPPH showed IC_50_ values in ethanol (0.39 mg/mL) and water (0.33 mg/mL), while in H_2_O_2_, IC_50_ = 34.51 μg/mL in ethanol and 32.67 μg/mL in water	[64]
	Seeds	H_2_Cl_2_/methanol	In vitro	IC_50_ = 8.51 μg/mL	[140]
	Ripe fruit	-	In vitro	IC_50_ = 33.75 ± 2.23 mg (superoxide radicals); 8.21 ± 1.30 mg (hydroxyl radicals); 54.39 ± 2.40 mg (hydrogen peroxide); 22.91 ± 3.76 (nitric oxide)	[58]
	Bark	n-hexane	In vitro	IC_50_ = 2.23 ± 0.75 μM (+)-dihydrokaempferol, 3.20 ± 0.71 μM (6-hydroxyflavanone), 4.71 ± 0.11 μM	[52]
	Lyophilized fruit juice powder	Water (250 and 500 mg/kg)	In vivo	↓ reactive oxygen species (ROS), ↓ reactive nitrogen species (RNS)	[94]
Analgesic and Anti-inflammatory activity	Leaves	Chloroform (400 mg/kg) and methanol (400 mg/kg)	In vivo	Chloroform ↑ analgesic effect	[14]
	Leaves	Water (200 and 400 mg/kg) and gold nanoparticles (4 mg/kg and 5 mg/kg)	In vivo	↓ free radical formation, ↓ paw edema	[38]
	Leaves	Ethyl acetate (122 μg/mL) and methanol (172.1 μg/mL)	In vitro	↓ 5-LOX, ↓ PLA_2_	[82]
	Bark	Methanol (400 mg/kg body weight)	In vivo	↓ histamine, ↓ serotonin, ↓ prostaglandins, ↓ paw volume	[81]
	Fruits	Ethanol	In vivo	↓ inflammation, ↓ NF-κβ, ↓ IL-6, ↓ IL-8, ↑ IL-10, ↑ IL-4	[80]
Hypolipidemic	Leaves and pulp juice	-	In vivo	↓ glycemic index, ↓ total cholesterol (TC), ↓ triglycerides (TGs), ↓ body weight	[90]
	Fruit	-	In vivo	↓ LDL-cholesterol, ↓ insulin, ↓ leptin, ↓ TC, ↓ TGs, ↓ phospholipids, ↓ body weight (BW), ↓ adipose tissue lipid deposits	[141]
	Leaves	Ethanol	In vitro	↓ α-glucosidase activity, ↑ insulin secretion	[49]
	Fruit	Ethyl alcohol and aqueous extract	In vivo	↓ blood glucose levels	[110]
Gastroprotective	Bark	Ethanol (200 mg/kg BW)	In vivo	↓ MPO activity in ulcerative colitis	[126]
	Fruit	Water and chloroform extract	In vivo	↓ diarrhea, ↓ IL-18	[124]
Anti-aging	Fruit pulp	Ethanol (140 μg/mL)	In vitro	↓ collagenase, ↓ elastase activity↑	[136]
	Emulgel formulation from fruit extract	-	In vitro	↑ photoprotection ability, ↑ quenching effect	[137]
Anti-arthritic	Leaf	Ethanol (400 mg/kg)	In vivo	↓ paw edema, ↓ protein denaturation	[38]

Abbreviations: DPPH: 2,2-diphenyl-1-picrylhydrazyl, IC_50_: half maximal inhibitory concentration, EC_50_: half maximal effective concentration, ↓: decrease, ↑: increase, 5-LOX: 5-lipoxygenase, PLA_2_: phospholipase A_2_, NF-κβ: nuclear factor kappa β, IL-6: interleukin-6, IL-8: interleukin-8, IL-10: interleukin-10, IL-4: interleukin-4, MPO: myeloperoxidase, IL-18: interleukin-1.

## Data Availability

No new data were created or analyzed in this study.

## Abbreviations

T_2_DM	Type 2 diabetes mellitus
MeSH	Medical subject heading
SOD	Superoxide dismutase
CAT	Catalase
GSH	Glutathione
GPx	Glutathione peroxidase
O2–	Superoxide
H2O2	Hydrogen peroxide
OH°	Hydroxyl
NO	Nitric oxide
GSK-3β	Glycogen synthase kinase 3β
CK1	Casein kinase 1
IL	Interleukin
LPO	Lipid peroxidation
COX-2	Cyclooxygenase-2
TNF-α	Tumor necrosis factor-α
TC	Total cholesterol
TGs	Triglycerides
LDL	Low-density lipoprotein
CVDs	Cardiovascular disorders
ROS	Reactive oxygen species
RNS	Reactive nitrogen species
GERD	Gastroesophageal reflux disorder
BW	Body weight
SIBO	Small intestinal bacterial overgrowth
PG	Prostaglandin
IBD	Inflammatory bowel disease
UC	Ulcerative colitis
MPO	Myeloperoxidase
MDA	Malondialdehyde
GIT	Gastrointestinal tract
RA	Rheumatoid arthritis
ALT	Alanine transaminase
AST	Aspartate transaminase
ALP	Alkaline phosphatase
UV	Ultraviolet
WHO	World Health Organization
ICH	International Council for Harmonization of Technical Requirements for Pharmaceuticals for Human Use
FDA	Food and Drug Administration
NIH	National Institutes of Health
EMA	European Medicines Agency
EFSA	European Food Safety Authority
MHRA	Medicines and Healthcare products Regulatory Agency
NNHPD	Natural and Non-prescription Health Products Directorate
NMPA	National Medical Products Administration
PMDA	Pharmaceuticals and Medical Devices Agency
DRAP	Drug Regulatory Authority of Pakistan
LD	Lethal dose
RTS	Ready-to-serve
